# Effects of resource availability and interspecific interactions on Arctic and red foxes' winter use of ungulate carrion in the Fennoscandian low‐Arctic tundra

**DOI:** 10.1002/ece3.11150

**Published:** 2024-04-01

**Authors:** Simon Lacombe, Rolf Ims, Nigel Yoccoz, Eivind Flittie Kleiven, Pedro G. Nicolau, Dorothee Ehrich

**Affiliations:** ^1^ Department of Arctic and Marine Biology UiT the Arctic University of Norway Tromso Norway; ^2^ Département de Biologie Ecole Normale Superieure de Lyon Lyon France; ^3^ Norwegian Institute for Nature Research Tromso Norway

**Keywords:** Arctic fox (*Vulpes lagopus*), intraguild interactions, occupancy, red fox (*Vulpes vulpes*), resource availability, scavengers, tundra

## Abstract

In the Arctic tundra, predators face recurrent periods of food scarcity and often turn to ungulate carcasses as an alternative food source. As important and localized resource patches, carrion promotes co‐occurrence of different individuals, and its use by predators is likely to be affected by interspecific competition. We studied how interspecific competition and resource availability impact winter use of carrion by Arctic and red foxes in low Arctic Fennoscandia. We predicted that the presence of red foxes limits Arctic foxes' use of carrion, and that competition depends on the availability of other resources. We monitored Arctic and red fox presence at supp lied carrion using camera traps. From 2006 to 2021, between 16 and 20 cameras were active for 2 months in late winter (288 camera‐winters). Using a multi‐species dynamic occupancy model at a week‐to‐week scale, we evaluated the use of carrion by foxes while accounting for the presence of competitors, rodent availability, and supplemental feeding provided to Arctic foxes. Competition affected carrion use by increasing both species' probability to leave occupied carcasses between consecutive weeks. This increase was similar for the two species, suggesting symmetrical avoidance. Increased rodent abundance was associated with a higher probability of colonizing carrion sites for both species. For Arctic foxes, however, this increase was only observed at carcasses unoccupied by red foxes, showing greater avoidance when alternative preys are available. Supplementary feeding increased Arctic foxes' carrion use, regardless of red fox presence. Contrary to expectations, we did not find strong signs of asymmetric competition for carrion in winter, which suggests that interactions for resources at a short time scale are not necessarily aligned with interactions at the scale of the population. In addition, we found that competition for carcasses depends on the availability of other resources, suggesting that interactions between predators depend on the ecological context.

## Graphical abstract

We used a 16‐year‐long camera trap survey to analyze the interactions between Arctic and red foxes at supplied carrion in the Fennoscandian low‐Arctic tundra. We found that competition for carcasses depends on the availability of other resources, suggesting that interactions between predators depend on the ecological context.

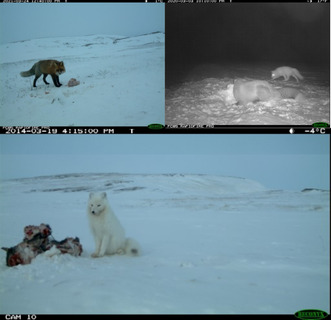

## 1 INTRODUCTION

In extreme environments, endemic species display a wide range of adaptations enabling them to cope with harsh climates and low productivity (Paine, 1980; Scholander et al., 1950). These adaptations, combined with low species diversity, often result in a low competitive ability (Goldberg & Novoplansky, 1997; Lindstedt & Boyce, 1985). The endemic biodiversity of Earth's most extreme ecosystems is therefore highly sensitive to species invasions, which can occur when one or more environmental stressors are relaxed (Archer & Predick, 2008; Walther et al., 2009).

Low Arctic tundra is characterized by a cold climate and a short growing season, resulting in a low productivity (Callaghan et al., 2004). Food webs are relatively simple and consist in tri‐trophic networks, with a guild of predators specializing to various degrees on herbivorous small rodents (Ims et al., 2017; Killengreen et al., 2007). These trophic networks are affected by important fluctuations in resource availability at both seasonal and multi‐annual scales. Indeed, interruption of the growing season and harsh weather conditions cause the abundance of resources for predators to drastically decline during the winter (Aars & Ims, 2002; Johnsen et al., 2016). In addition, thick snow cover reduces the availability of rodents for predators (Lindström et al., 1994). Multi‐annual fluctuations, on the other hand, are driven by the cyclic population dynamics of voles and lemmings (Ims & Fuglei, 2005). To cope with these recurrent periods of food scarcity, most tundra predators have developed opportunistic feeding behaviors and rely on alternative food resources (Killengreen et al., 2011; Nater et al., 2021). In particular, many predators are also facultative scavengers (Gomo et al., 2020) and use ungulate carcasses (carrion) as additional resources during the winter, taking advantage of their rather high supplies of food and accessibility (Killengreen et al., 2011; Mattisson, Andrén, et al., 2011). Therefore, in many Arctic and boreal ecosystems, predator communities are impacted by availability of ungulate carrion, which has been shown to affect predator breeding (Ehrich et al., 2017; Mattisson, Andrén, et al., 2011) and winter survival (van Dijk et al., 2008), potentially impacting their geographical range (Henden et al., 2014; van Dijk et al., 2008). Ungulate carcasses represent localized resources that may attract several scavengers, acting as a hot‐spot for interactions—both interspecific and intraspecific—in an otherwise low‐density environment (Henden et al., 2014). For instance, in Northern Sweden, wolverines (*Gulo gulo*) and lynxes (*Lynx lynx*) often share the same carcasses (Mattisson, Andrén, et al., 2011; Mattisson, Persson, et al., 2011), while in the Canadian boreal forests, wolves (*Canis lupus*), black bears (*Ursus americanus*), coyotes (*Canis latrans*), and Canadian lynxes (*Lynx canadensis*) all use wolf‐killed carcasses (Tattersall et al., 2020). Still, the way species interact at these carcasses is poorly known, especially in the Arctic, and likely depends on the species and ecological context. Understanding how winter use of carrion is impacted by interspecific competition is crucial for a better understanding of the winter dynamics of Arctic predator communities.

The Fennoscandian tundra is home to a diverse community of facultative scavengers that includes two canid species: the Arctic fox (*Vulpes lagopus*) and the red fox (*Vulpes vulpes*) (Ims et al., 2017). Although the red fox is a temperate species less adapted to the conditions of the Arctic, the recent increase in the availability of carcasses from semi‐domestic reindeer (*Rangifer tarandus*) (Henden et al., 2014; Ims et al., 2017), combined with indirect effects of a warmer climate and other anthropogenic factors, led to an increase in their density in the low Arctic and alpine tundra of Fennoscandia (Hersteinsson & MacDonald, 1992; Killengreen et al., 2007). On the contrary, the Arctic fox population reached critically low levels during the 20th century, facing near extinction in the beginning of the 21st century (Angerbjörn et al., 2013), and the species is now considered endangered in Fennoscandia (Angerbjörn & Tannerfeldt, 2014; Berteaux et al., 2017). This decline has been attributed to two main drivers: a climate related disturbance of lemming cycles (Ims et al., 2011, 2017) and increased competition with red foxes (Elmhagen et al., 2017; Hersteinsson & MacDonald, 1992). Consistent with the competition‐hypothesis, several recent studies have found that red foxes limit Arctic foxes' habitat use at a year‐to‐year scale (Hamel et al., 2013; Rød‐Eriksen et al., 2023), revealing that Arctic fox populations are highly sensitive to the presence of red foxes. Still, where the two species co‐occur, little focus has been put on their interactions at a short temporal scale (e.g., from day to day). In particular, how their winter use of reindeer carcasses is impacted by interspecific competition remains unknown. As shown for dens (Tannerfeldt et al., 2002), it is possible that red foxes tend to monopolize carcasses, preventing Arctic foxes from accessing them. When other resources are available, Arctic foxes' reliance on carrion is relatively low (Ehrich et al., 2015; Elmhagen et al., 2002; Killengreen et al., 2011) and avoiding carcasses used by a competitor may be the best compromise to minimize risks. In years with scarcity of live prey however, reliance on carrion is important and Arctic foxes may be forced to risk encounters. Competitive dominance of red foxes is nonetheless not universal, and the outcome of the interactions between the two species seems highly context dependent. For instance, in several places across the Canadian Arctic tundra, red foxes do not affect Arctic fox home‐range size, den occupancy, or access to resources (Gallant et al., 2012; Lai et al., 2022). Although all these regions also belong to the Arctic tundra biome, the ecological conditions differ from Northern Fennoscandia in various aspects: climate is colder and access to anthropogenic resources is also lower, reducing overall productivity. Tougher conditions may thus relax competition between the two species due to red foxes' higher energy requirements and lower adaptation to cold temperatures and food scarcity (Hersteinsson & MacDonald, 1982, 1992). Therefore, although red foxes are competitively dominant in Fennoscandia, this dominance could be relaxed during the winter when the conditions get more extreme, reducing their ability to monopolize resources.

In this study, we investigated how foxes' use of carrion in winter is impacted by interspecific interactions and availability of other food resources. Using a 16‐year long camera trap survey, we focused on the interactions between Arctic and red foxes at supplied carrion in the Varanger peninsula, at the western fringe of the Eurasian Arctic tundra. In line with the known competitive interactions between the two species, we predicted that (1) presence of red foxes would limit Arctic foxes' use of carrion. We also predicted that (2) the outcome of competition for carrion would depend on the availability of alternative food resources such as small rodents, with Arctic foxes risking encounters with red foxes to a lesser extent on years when other resources are abundant. We used a multi‐species dynamic occupancy model to estimate use of carrion by the two species at a weekly scale, while accounting for the imperfect detection process inherent to camera trap surveys (Kery & Royle, 2020; MacKenzie et al., 2017).

## 2 MATERIALS AND METHODS

### 2.1 Study area

**FIGURE 1:**
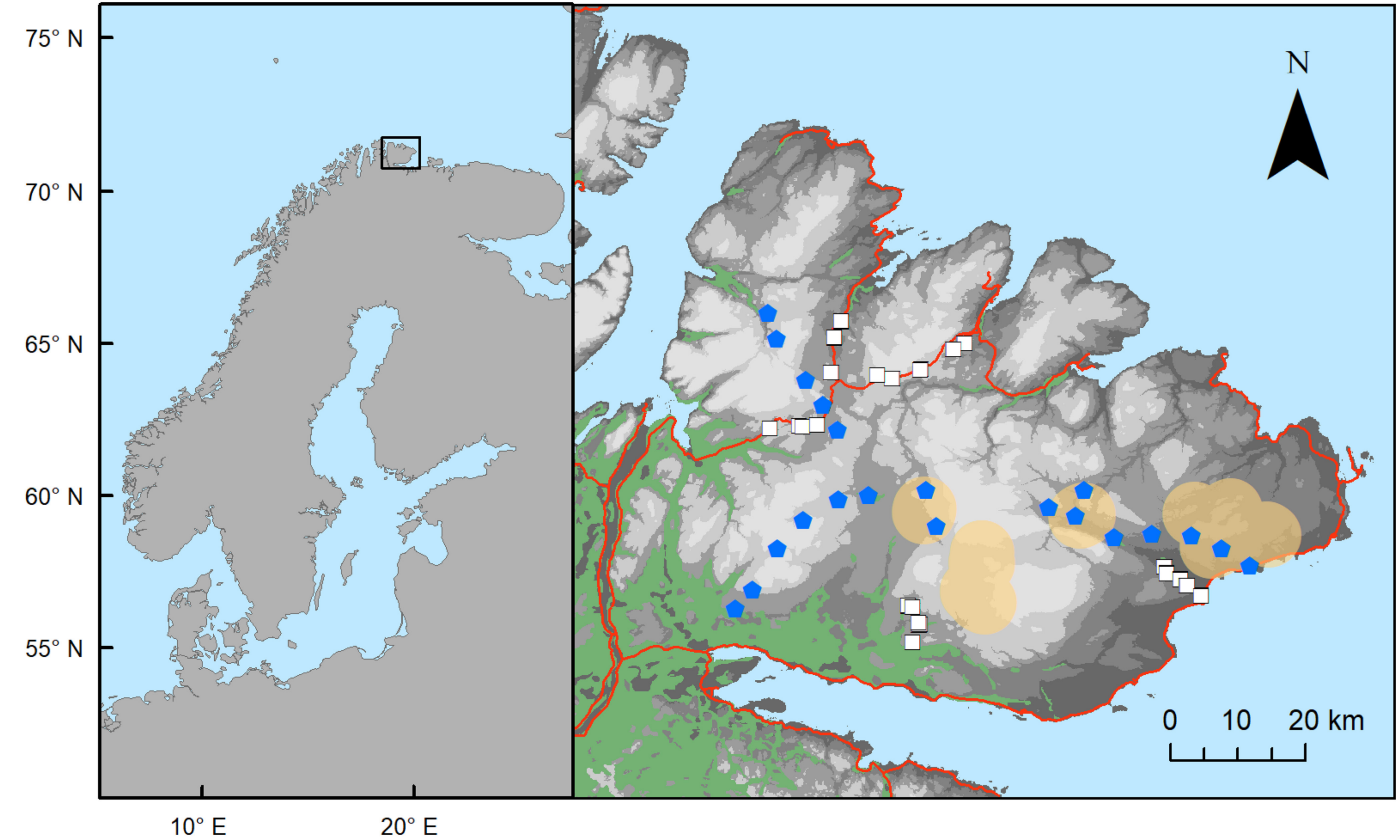
Location of the Varanger Peninsula in northern Norway and map of the study area. Camera‐trap sites are shown with blue polygons and locations of small rodent trapping sites with white squares. The approximate location of feeding stations is shown with yellow circles. Roads are shown in red and forests in green. The altitude is represented in shades of gray, with darkest tones indicating low altitudes. The gradations are every 100 m.

The Varanger peninsula (70–71° N, 29–31° E) is located in north‐eastern Norway, in the western part of the Eurasian Arctic tundra. The peninsula is characterized by steep climatic gradients related to altitude and distance from coast (Ims et al., 2017) (Figure 1). The south‐west of the peninsula is mostly covered with sub‐Arctic mountain birch forest (*Betula pubescens*), while the north‐east and the interior highlands are made up of more sparse tundra vegetation. Available prey to both fox species are small rodents (tundra vole, *Microtus oeconomus*; gray‐sided vole, *Myodes rufocanus* and Norwegian lemming, *Lemmus lemmus*), mountains hares (*Lepus timidus*), and ptarmigans (*Lagopus* spp.). In addition, the area is used as pasture for semi‐domestic reindeers (*Rangifer tarandus*), and reindeer carrion represent an additional food supply. Finally, the coastal habitats can provide important resources, due to the high productivity of the surrounding ice free marine ecosystems and the anthropogenic subsidies from the human settlements (Killengreen et al., 2011). Besides Arctic and red foxes, the facultative scavengers likely to consume ungulate carcasses in the region are wolverines, golden eagles (*Aquila chrysaetos*), white‐tailed eagles (*Haliaeetus albicilla*), and common ravens (*Corvus corax*).

Since 2005, the Varanger Peninsula is part of the Arctic fox conservation program of the Norwegian Environment Agency. This conservation plan consisted of two main phases. Firstly, a red fox culling operation started in 2005 to relax the competition pressure on the Arctic fox and resulted in 3894 red foxes being culled between 2005 and 2021. Still, this was not sufficient to enable proper recovery of the Arctic fox population (Ims et al., 2017) and the conservation program was taken further in 2017 with supplementary feeding and reintroduction of captive bred individuals. In this context, 20 feeding stations for Arctic foxes have been deployed and 65 captive bred juvenile Arctic foxes have been reintroduced between 2018 and 2020. The entrance of the feeding stations was dimensioned to allow Arctic foxes to enter while being too small for the larger red foxes (Thierry et al., 2020). The use of the feeding stations was monitored with camera traps, which confirmed that they were used nearly exclusively by Arctic foxes. Overall, a total of 4.6 tons of dog pellets, accessible to Arctic foxes only, were used at the different stations (Ehrich & Ims, 2021), creating an interesting example of additional resource available only to the subdominant competitor. Taken together, these measures triggered an important increase in the Arctic fox population, resulting in the minimum population size estimated from genetic capture‐mark‐recapture increasing from 1 to c.a. 25 between 2017 and 2021 (Ulvund et al., 2021).

### 2.2 Sampling design

**FIGURE 2:**
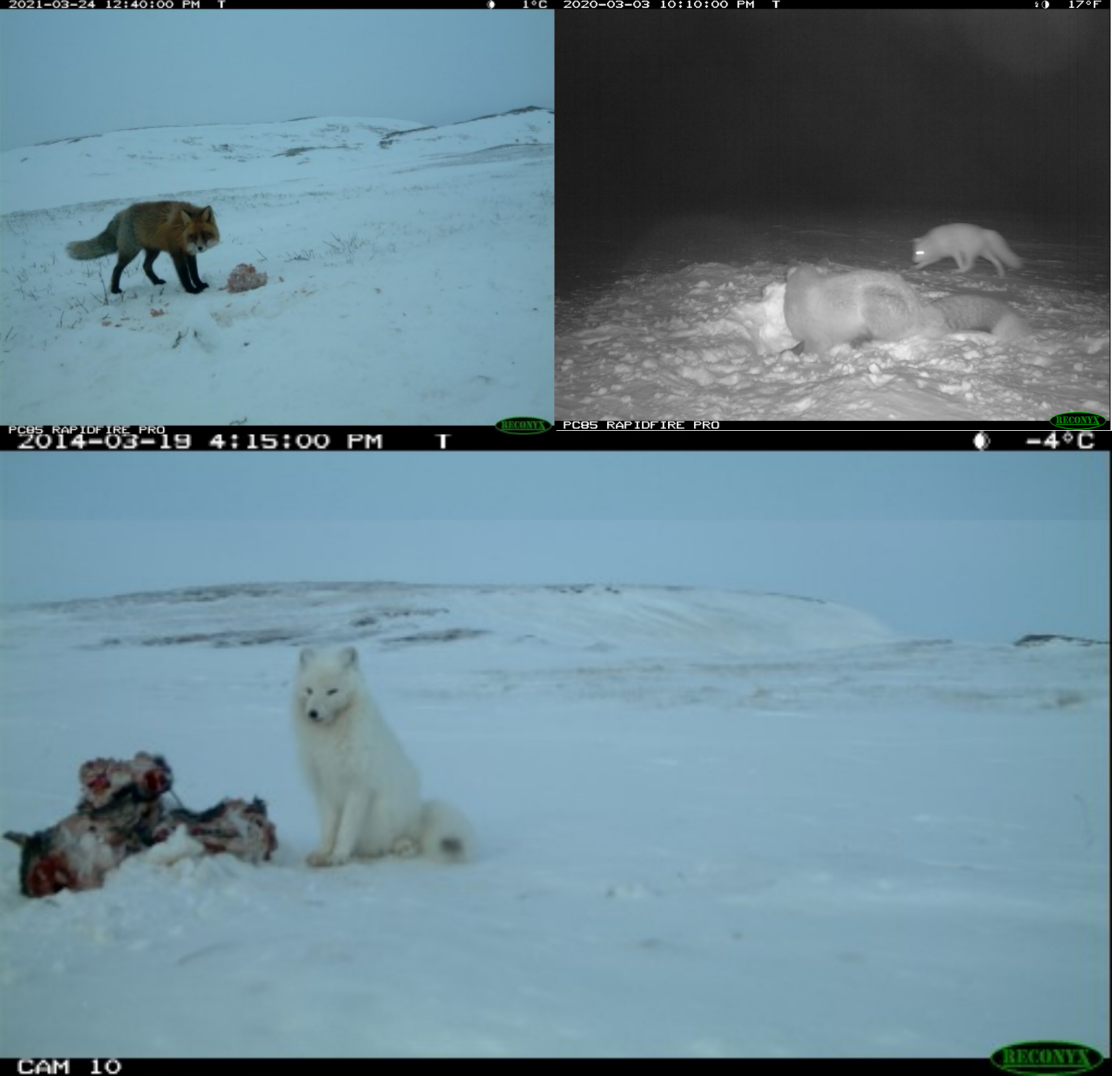
Example of images from the camera‐trap survey on Varanger Peninsula. The images show, from top left to bottom, a red fox, Arctic and red foxes (red fox in the foreground and Arctic fox in the background), and an Arctic fox. Frozen remains of reindeer were placed in front of the cameras and are visible in the top right and bottom pictures.

The camera trap survey was initiated in 2005, but as no pictures of Arctic foxes were obtained that year, the sampling period used for this study covered 16 years, from 2006 to 2021. In each year, between 16 and 20 camera traps were active taking photos every 10 min for 2 months in late winter (Figure 1). We used several camera models with different fields of view (Camtrak; Reconyx Rapidfire, Hyperfire and Hyperfire 2). The cameras were painted in white, modified to have a flat front keeping snow from accumulating and powered by external batteries placed in a waterproof container under the snow. Pictures were visually inspected and presence of red and Arctic foxes was recorded. Pictures with bad visibility were excluded. To estimate the use of carrion, a block of ca 15 kg of frozen reindeer slaughter remains (originally produced as dog food and consisting of tendons, fat, small entrail, and meat fragments) was placed 2–3 m in front of each camera and replaced two to three times during the season. For each photo, we recorded whether the carcass was present. Images of the two species from the camera‐trap survey are visible on Figure 2.

To account for environmental variability, we measured elevation (range: 50–410 m), distance to coast (0.3–27.9 km), distance to road (0.3–22.0 km) and distance to forest (0.0–11.3 km) at the locations of the cameras. We also evaluated the proportion of productive habitats within a 5‐km radius (0.0%–66%). Using a vegetation map of Finnmark (Johansen et al., 2009), we defined productive habitats as areas covered by forest or by the most productive heath class comprising erect shrubs. Because some variables were correlated, we performed principal component analysis on these five geographical covariates and used the two first axes (explaining respectively 39.7% and 27.5% of the variation, Figure S4) as proxies for two gradients: the first axis correlated with the distances to roads and coastline, and with the elevation. We interpreted it as a gradient from coastal to inland environments (hereafter CLG, with positive values indicating inland environments). The second axis correlated with the distance to forest and the proportion of productive areas and was interpreted as a tundra to forest gradient (TFG, with positive values for sites close to forest environments).

We considered the availability of two main food resources other than the artificial carcasses: small rodents and supplemental feeding (dog pellets). We used an index of rodent abundance from a rodent monitoring program (number of trapped individuals per 100 trap‐nights). Briefly, this index is based on the number of rodent individuals trapped during a 2‐day survey twice a year (see Ims et al., 2011 for more details). We used fall abundance from the fall preceding the winter camera trapping of three rodent species: tundra vole, gray‐sided vole, and Norwegian lemming. We averaged the abundances across all trapping sites on the Varanger peninsula to obtain an annual index (see Figure 1 for spatial distribution of trapping sites). Although this is not a precise quantification of the small rodent abundance available to the foxes at the time of the camera survey (the timing of the decline from fall to winter can vary, and snow depth and hardness can impact hunting efficiency) it does provide information about general level of availability of this resource at the scale of the peninsula.

To evaluate the effects of supplemental feeding, we calculated a feeding station density index for each camera trap. To do so, we used the locations and start dates of the 20 feeding stations and built a time‐dependent kernel density estimator, accounting for the start date of each feeding station. We set the spatial resolution to 2 km and the bandwidth to 15 km, to roughly match with the estimated home range sizes of Arctic foxes (Lai et al., 2016). Then, we extracted the value of the kernel density estimator for each camera × year combination.

### 2.3 Occupancy modeling

**FIGURE 3:**
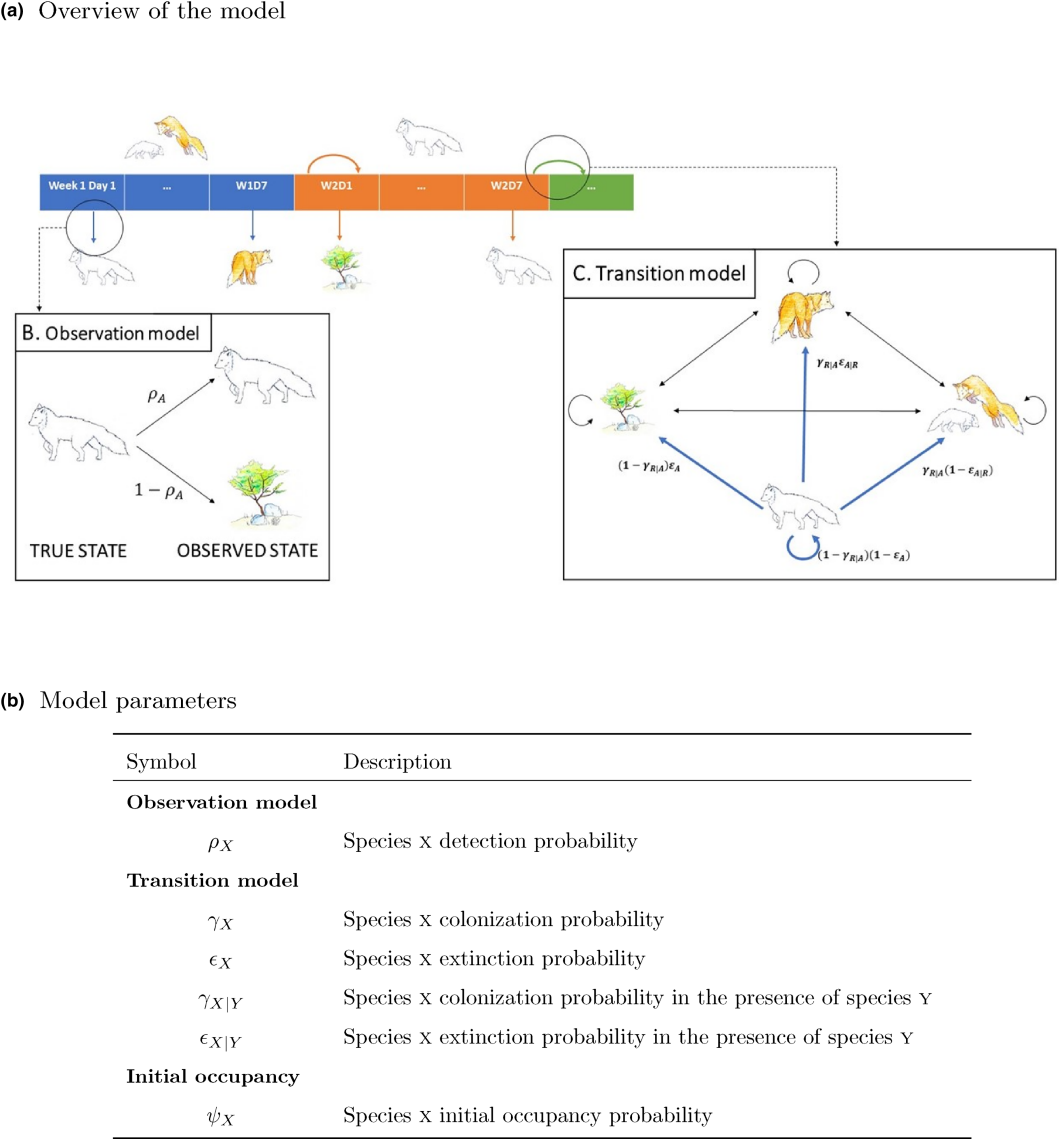
Presentation of the multi‐species dynamic occupancy model used in this study: (a) Overview of the model. Panel A presents a state sequence at a camera station a given year. Primary periods of 1 week are shown with various colors (blue: week 1, orange: week 2, green: week 3, …) and the observed state is shown under the state sequence. The occupancy state is constant during each week and the observed state can vary every day based on the observation model (panel B). The transition model (panel C) describes how occupancy states vary between weeks; (b) Presentation of the model parameters.

We modeled Arctic and red foxes use of carrion by fitting a two‐species dynamic occupancy model adapted from Fidino et al. (2018) (Figure 3 and Appendix S1). Because the camera stations are baited with artificial carcasses, occupancy does not here simply refer to species presence/absence in the landscape but rather to the use of carrion, which is impacted by both resource use, and local abundance of the target species (Stewart et al., 2019).

We first summarized Arctic and red foxes presence or absence on the pictures to daily occurrence. A sufficient number of pictures was needed to provide reliable information about the presence/absence of a species on a given day. Therefore, we removed the days with less than 36 pictures out of the daily expected 144 pictures for each site, such that every day had at least 25% of the maximum number of pictures. To obtain information on how fox species interact on a short time scale, we focused on the dynamics within a winter, treating each winter as separate independent replicates. Hence, every camera × year combination was included in the model as an independent time‐series, accounting for a total of 315 camera winters. Each time series was then segmented into primary periods of 1 week starting from the day when the carrion was introduced for the first time. A week was included in the analysis if it had more than 3 days of observations. We kept camera × year combinations that had more than 3 weeks of data (*n* = 288), and systematically removed all observations after 7 weeks, to match with the period of activity of most cameras. We assumed occupancy to remain constant during primary periods (assumption hereafter referred as the “closure assumption”) with four possible states: 0, no species; *A*, Arctic foxes only, *R* red foxes only, and *AR* both fox species. Between weeks, the occupancy states could vary based on species colonization probabilities (*γ*_*x*_ – probability that a site unoccupied by species *x* is occupied the following week, Figure 3) and extinction probabilities (*ϵ*_*x*_ – probability that a site occupied by species *x* is abandoned the following week, Figure 3). Because we could not apply these probabilities for the first week, we defined species‐specific initial occupancy probabilities (*ψ*_*x*_ – probability that species *x* is present at a site the first week). Although the occupancy state was deemed constant during each week, the observed state at a camera could vary between days based on species‐specific detection probabilities (*ρ*_*x*_ – probability that species *x* is observed at a camera when present, Figure 3).

Our choice of primary periods of 7 days resulted from a trade‐off between two conflicting constraints. On the one hand both fox species have large home ranges and can cover great distances every day (Alexandra et al., 2002). Therefore, a too long primary period would cause serious violation of the closure assumption. On the other hand, our model is largely based on the estimation of detection probabilities, which requires enough observations to be accurately estimated. In fact, Kery and Royle (2020) suggested a minimum of five observations per primary period to obtain reliable estimates.

To account for environmental and seasonal variability, we included covariates in the model using the logit link function. Covariate selection was based on ecological plausibility rather than model selection criteria, which may be inadequate for this kind of hierarchical model (Carrillo‐Rubio et al., 2014). We modeled the detection probabilities as functions of the presence of the carrion, as the carrion on some occasions was removed or eaten up likely affecting the probability of animals entering the detection zone of the camera traps (although small fragments and smell usually remained). When modeling occupancy, accounting for the major sources of potential variability in the detection probabilities is important (Kery & Royle, 2020). Therefore, we also used the categorical variable *year* as a random effect on the detection probability to summarize the seasonal variability (e.g., due to weather or availability of natural reindeer carcasses) not accounted for by our covariates. Colonization, extinction, and initial occupancy probabilities were modeled as functions of the geographical covariates (*FTG* and *CLG*) (Hamel et al., 2013; Killengreen et al., 2011), rodent abundance (Elmhagen et al., 2017; Ims et al., 2017) and the feeding stations proximity index. We also ran an alternative model with a categorical covariate before 2018–after 2018 to account for the release of captive bred individuals at the regional scale. This model resulted in a high negative correlation between effects of feeding stations and reintroduction (e.g. *R* ^2^ = 0.43 for Arctic foxes colonization probability), showing that these two covariates had a similar effect on occupancy, and therefore suggesting we could not disentangle the effects of reintroduction and supplementary feeding. Thus, we removed the before 2018–after 2018 covariate from the model and assumed the supplementary feeding index to summarize both changes in numbers and supplementary feeding. This seems appropriate as the new individuals were released on dens with feeding stations and are expected to mostly use these territories. In addition, because initial occupancy, colonization, and extinction probabilities are likely to be affected by other factors not accounted for in our model, we also included *year* as a random effect to account for other sources of variations (e.g., yearly variations in both species' abundance). We considered adding a site random effect to the detection, colonization, and extinction probabilities to account for the fine‐scale location of the carrion sites, as factors like proximity to breeding dens or snow depth (that can vary greatly locally and affect availability of rodents for predators) could affect carrion use and detectability. However, this random effect did not improve the fit of the model, and greatly increased the number of parameters. Therefore, we decided to not keep the site random effect in the model.

Finally, colonization and extinction probabilities were modeled as functions of the presence of the competitor in either the considered or the next time steps. In order to estimate the effect of resource availability on how species compete for carrion, we allowed the effects of competition on colonization and extinction to vary with the amount of supplemental feeding and with rodent abundance. We centered and standardized all continuous covariates to be able to compare the estimated effect sizes.

### 2.4 Bayesian implementation

We fitted our model under the Bayesian framework with Markov Chain Monte Carlo (MCMC) methods using JAGS 4.3.0 (Plummer, 2003) and the package runjags (Denwood & Plummer, 2022) under R 4.0.3 (R Core Team, 2020). Four MCMC chains were run in parallel with an adaptation phase of 1000 iterations and a burn‐in phase of 10,000 iterations. The posteriors were then sampled 25,000 times with a thinning rate of one in five, yielding a total of 20,000 samples of the posterior distribution. Priors for logit‐linear intercepts and slopes were assumed to follow a Logistic(0,1) distribution as suggested in Fidino et al. (2018), and priors for variance of the random effects were assumed to follow a uniform distribution. We also derived overall carrion use from the model estimates, which we defined as the stationary occupancy probabilities. To do so, we used the transition matrix obtained from colonization and extinction probabilities, and calculated its steady state using the R package markovchain (Spedicato et al., 2021).

We checked model convergence by visually inspecting the trace plots and by calculating the Gelman and Rubin's R statistic (Brooks & Gelman, 1998). To evaluate how the observation and the transition parts of the model fit the data, we performed a posterior predictive check (Carrillo‐Rubio et al., 2014). Briefly, we simulated 10,000 datasets using the model estimates and calculated Bayesian *p*‐values for the detection model and the transition model. Bayesian *p*‐values are defined as the proportion of times the observed dataset fitted the model better than the simulated one (see Appendix S2). The model is assumed to have a proper fit when the Bayesian *p*‐value is between .1 and .9 (Kery & Royle, 2020).

## 3 RESULTS

### 3.1 Model performance

Both the Gelman–Rubin statistic (≤1.05 for each parameter) and the trace plots indicated model convergence. Regarding the goodness‐of‐fit test, we obtained mixed results: the Bayesian *p*‐value for the latent part of the model indicated adequate model fit (Bayesian *p*‐value = .34, Figure S1). For the observation part, it indicated a systematic lack of fit (Bayesian *p*‐value = 0, Figure S1). This is expected to happen for mobile species (likely violating the closure assumption), or when there are unmodeled sources of variation in detection probabilities (Kery & Royle, 2020). In our case both phenomena are likely. It was hence difficult to fully account for non‐detection of these species, which is important to keep in mind when interpreting the following results. Plotting the chi‐squared residuals did not allow us to link the lack of fit to any species or site in particular (Figure S2).

### 3.2 Arctic and red foxes' average use of carrion

**FIGURE 4:**
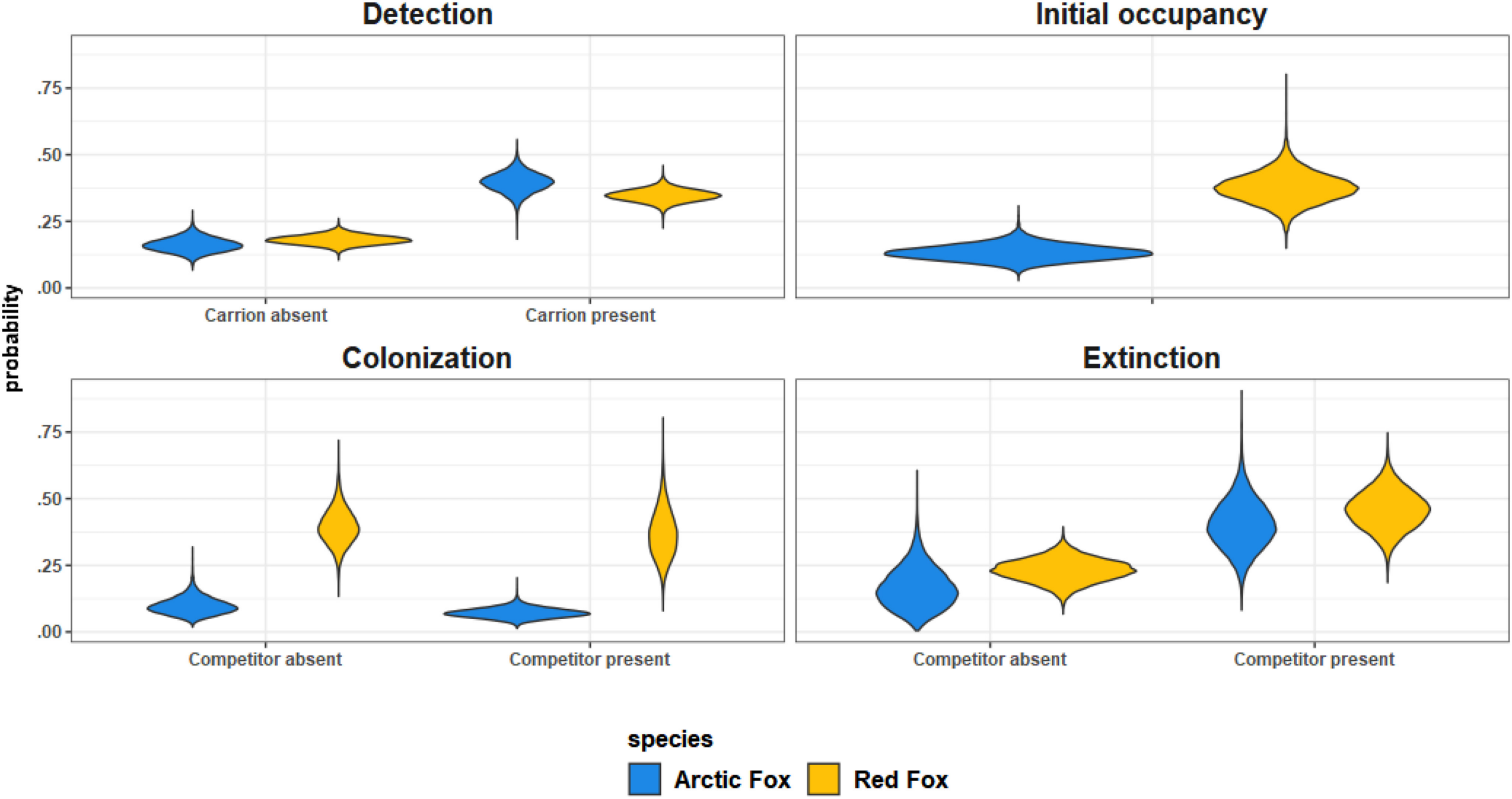
Posterior distributions of average detection, initial occupancy, colonization and extinction probabilities for Arctic (blue) and red (yellow) foxes. All probabilities are calculated using the estimated intercept only, setting the value of covariate to their average value across the dataset.

Out of a total of 8901 camera days, red foxes were detected at the carrion sites on 1326 days and Arctic foxes on 556. They were detected together on 92 camera days. The other predators seen at the carrion were wolverines (316 days), golden eagles (420 days), white‐tailed eagles (115 days), and common ravens (5742 days). The bait was present in front of the camera on 5490 days, accounting for c.a. 62.7% of all camera‐days. After introduction, the bait remained present for 22.3 ± 1.4 days (mean ± 95% confidence interval). Even after the main carrion block disappeared, small fragments usually remained still attracting predators to inspect this location. Posterior distributions for all logit‐linear parameters and slope as well as for the variance component of the random year effects are available on Figure S3. Average detection probabilities were similar between Arctic (median [90% credibility interval]: 0.40 [0.32–0.47]) and red (0.35 [0.30–0.39]) foxes (Figure 4). When the carrion was absent, the detection probabilities decreased to 0.18 [0.15–0.22] for red foxes and 0.16 [0.11–0.22] for Arctic foxes (Table 1).

**TABLE 1:** Overview of estimated effects on Arctic and red fox occupancy processes.

Parameter	Symbol	CLG	TFG	Rodent abundance	Supplementary feeding	Competition	Competition × rodent ab.	Competition × supp. Feeding	Carcass	Random year variation
Initial occupancy	*ψ*	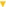	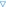	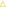	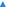	×	×	×	×	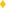
Colonization probability	*γ*	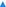	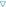	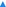 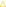	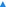		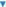		×	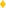
Extinction probability	*ϵ*				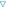 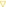	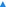 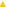	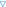		×	
Detection probability	*ρ*	×	×	×	×	×	×	×	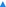 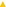	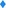 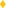

*Note*: ▴/▾: positive/negative effect with 90% credible interval not overlapping 0. ∆/∇: positive/negative effect with 70% CI not overlapping 0. ♦: Variance component with posterior distribution separate from 0. Blue: Arctic fox, Yellow: Red Fox. ×: effects not considered in the model. When no symbol is present, the effect is indistinguishable from 0. Whenever we tested an effect (no × symbol) it was tested for both red and Arctic foxes. CLG and TFG are two geographical covariates, standing for coast to land gradient and tundra to forest gradient respectively.

Arctic foxes had average initial probability of carrion use of 0.13 [0.081–0.19] and they colonized carrion with a probability of 0.094 [0.047–0.17]. These probabilities were in both cases lower than for red foxes (0.37 [0.27–0.49] and 0.39 [0.27–0.54] respectively) (Figure 4). Arctic foxes had a lower extinction probability than red foxes, although this difference was less pronounced than for colonization and initial occupancy, with an average extinction rate of 0.16 [0.040–0.34] for Arctic foxes and 0.23 [0.14–0.31] for red foxes (Figure 4).

### 3.3 Effect of geographical variability on use of carrion

**FIGURE 5:**
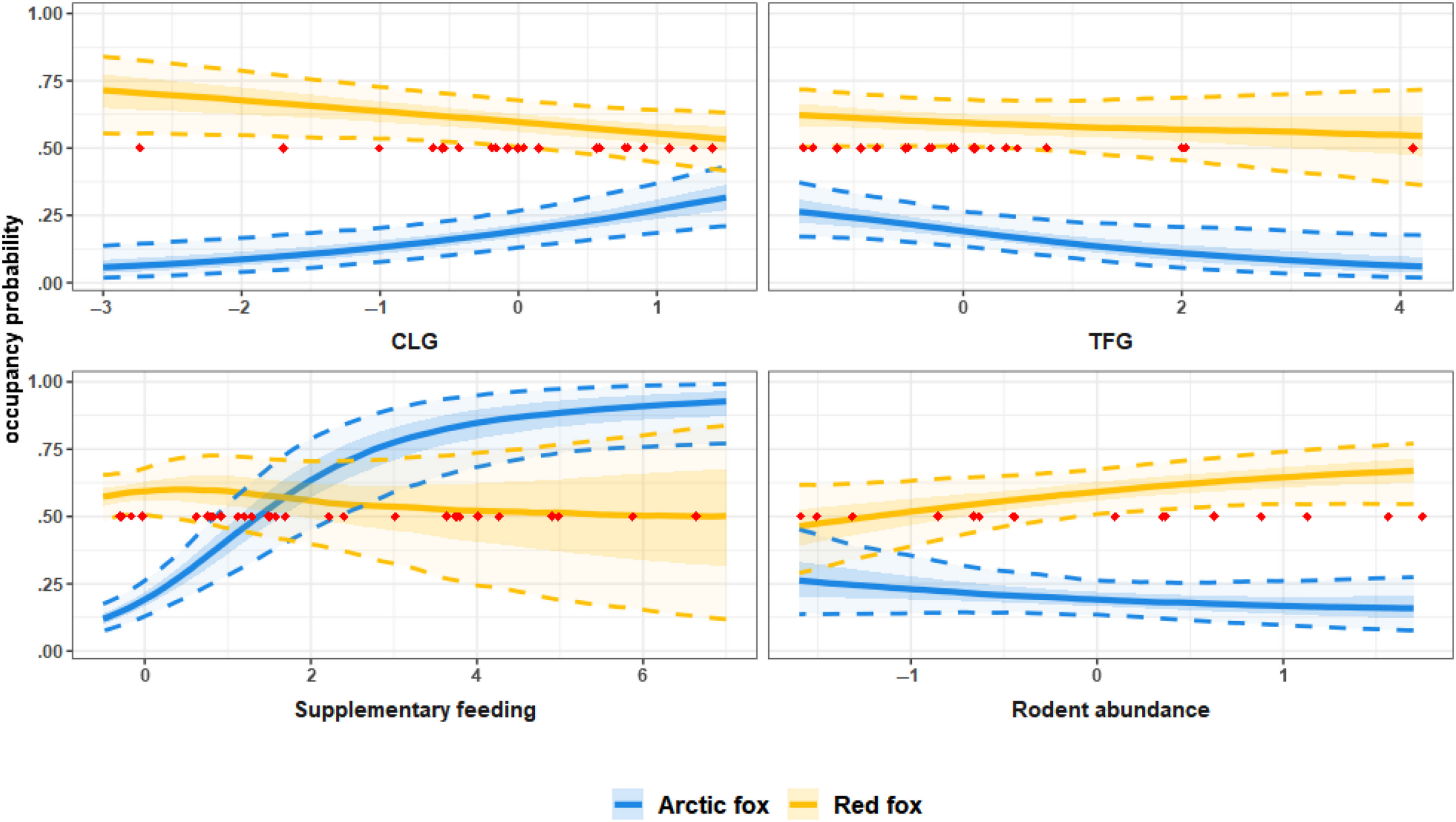
Probability of carrion use for Arctic (blue) and red (yellow) foxes as a function of the positions on the coast to land (CLG) and tundra to forest (TFG) gradients, the supplementary feeding index and the rodent abundance. Solid lines represent posterior medians, shaded ribbons represent 50% and dashed lines 90% credible intervals. Red dots show the values taken by the covariate of interest in the real dataset. Carrion use is defined as the stationary occupancy probability, calculated as the steady state of the estimated transition matrix.

We found support for effects of the two geographical gradients on use of the carrion sites. Indeed, we found that Arctic foxes were more likely to colonize carrion farther from the coasts (Table 1—CLG). They also had lower colonization and initial occupancy probabilities closer to the forest than farther into the tundra (Table 1—TFG). Overall, this resulted in their probability of carrion use increasing from 0.057 [0.018–0.14] to 0.32 [0.21–0.43] as we move inland and decreasing from 0.26 [0.17–0.37] to 0.060 [0.019–0.18] approaching the forest (Figure 5). Red foxes had higher initial occupancy close to the coast (Table 1). Their use of carrion slightly decreased with the CLG gradient, from 0.71 [0.55–0.84] to 0.54 [0.42–0.63] (Figure 5), but it did not seem to be significantly affected by the TFG gradient.

### 3.4 Effects of competition and resource availability on use of carrion

**FIGURE 6:**
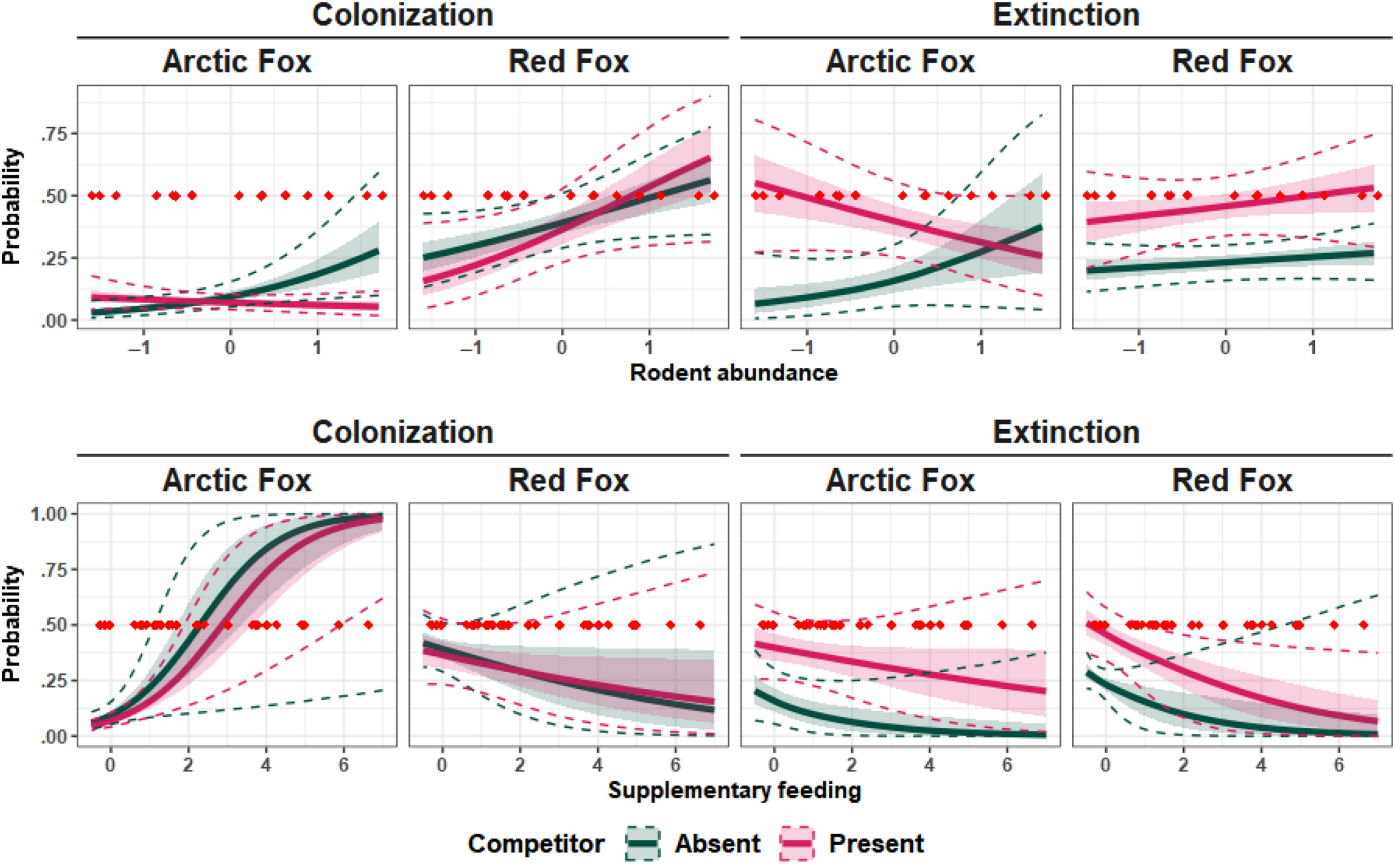
Arctic and Red foxes' colonization and extinction probabilities conditional to the other species' absence (green) or presence (magenta) as a function of rodent abundance and supplementary feeding. Solid lines represent posterior medians, shaded ribbons represent 50%, and dashed lines 90% credible intervals.

Rodent abundance had positive effects on both Arctic and red foxes' probability to colonize carrion sites (Table 1, Figure 6), but it did not strongly affect their overall carrion use (i.e., occupancy probability) (Figure 5).

Arctic foxes were more likely to start to use (colonize) carrion close to supplemental feeding stations (Table 1, Figure 6). They also had lower extinction probability and higher initial occupancy closer to feeding stations (Table 1, Figure 6). This led their probability of presence at carrion to be strongly affected by the amount of supplemental feeding, increasing from 0.12 [0.075–0.17] in sites without feeding to 0.93 [0.77–0.99] where feeding was most intense (Figure 5). As expected, red foxes' carrion use was not affected by supplementary feeding (Table 1, Figure 5).

For both species, we found that the probability to leave carrion more than doubled when the other species was present, increasing to 0.40 [0.23–0.59] for Arctic and to 0.46 [0.32–0.60] for red foxes (Table 1 and Figure 4). How carrion use was affected by interspecific competition did not depend on the amount of feeding (Table 1, Figure 6). Rodent abundance, however, had a negative effect on Arctic foxes' colonization probability, when red foxes were present, suggesting an increased avoidance of red foxes when rodents are more abundant (Table 1). This ended up canceling out the effect of rodents on Arctic foxes' colonization probability when red foxes were present (Figures 5 and 6). Abundance of rodents also seem to have decreased the extinction probability of Arctic foxes when red foxes were present (Table 1 and Figure 6), but the large uncertainty associated with extinction probability in years with high rodent abundance (Figure 6) and the lower statistical support for this effect (only the 70% CI does not overlap 0, Table 1) make it difficult to interpret it. This might be caused by the very low colonization probability of Arctic foxes in the presence of a red fox when rodents are abundant, leading to a low number of co‐occurrences, therefore limiting the estimation of their extinction probability in that situation.

## 4 DISCUSSION

In this study, we have been able to estimate Arctic and red foxes' presence at experimental reindeer carrion throughout a 16‐year survey on the Varanger peninsula using an occupancy modeling framework. It is important to keep in mind that, in our setup, the occupancy probability can be decomposed in two distinct probabilities: the probability that at least one individual is present in the area, which could be referred to as a strict occupancy probability (MacKenzie et al., 2004; Rota et al., 2016), and the probability that this individual uses the carrion (Lele et al., 2013; Stewart et al., 2019). Furthermore, in dynamic occupancy frameworks, occupancy estimates are known to reflect both abundance and movement rates at a broad geographical scale (Kate Broadley et al., 2019; MacKenzie et al., 2003). As all these aspects of occupancy cannot be distinguished from one another, occupancy probabilities must be interpreted in terms of both regional abundance, movement rate, and carrion attractiveness.

Throughout the study period, Arctic foxes' use of carrion remained low. Compared with red foxes, they had lower initial occupancy and colonization probabilities (Figure 4), resulting in an overall lower probability of occupancy (Figure 5). This likely reflects differences in abundance rather than in bait attractiveness, as red foxes were largely numerically dominant throughout most of the study (Ulvund et al., 2021). Arctic foxes in Fennoscandia have indeed suffered a drastic decline over the last century. The estimated population size on the Varanger Peninsula ranged between 21 (year 2009) and two individuals (2017) (Ims et al., 2017; Ulvund et al., 2021) until 2018 when 27 captive bred individuals were first reintroduced. Accurate estimates for red fox abundance in this area are not available, but c.a. 3800 red foxes have been culled on the peninsula in the study period (2005–2021; 42), supporting clearly higher numbers for this species. Contrary to our initial prediction, we did not find strong evidence for important asymmetric competition between Arctic and red foxes around carrion. Indeed, competition appeared to be mostly expressed through extinction rates, as Arctic and red foxes tended to leave carrion occupied by competitors to a higher extent. The effect of competition on extinction was similar between the two species, suggesting symmetrical avoidance behaviors (Table 1 and Figure 4). We also found that when rodent abundance was high, Arctic foxes had a higher probability of colonizing carrion sites. This was only true when the carrion was unoccupied by red foxes (Table 1 and Figure 6). Therefore, when rodents were most abundant, Arctic foxes' colonization probability was more strongly impacted by the presence of red foxes. In line with our second prediction, this suggests that the competitive interactions at carrion between the two species greatly depend on the availability of alternative food resources. Supplementary feeding also caused a very important increase use of carrion by Arctic foxes, with a probability of occurrence approaching 1 in sites close to many feeding stations (Figure 5), regardless of the presence of red foxes.

Several studies have documented red foxes to exclude Arctic foxes from breeding dens and resource patches, and it has been suggested that interference competition can lead to avoidance behaviors in Arctic foxes (Elmhagen et al., 2013; Hamel et al., 2013). However, in years with low rodent abundance, our results do not align with this hypothesis, as the two species tended to avoid each other to the same extent. This symmetrical avoidance may instead reflect the fact that tundra patches are unable to support large numbers of individuals (Lai et al., 2022). Another possible cause for these avoidance behaviors could be a quicker disappearance of the carrion—or of the parts most appealing to foxes—when other individuals are present. Further studies investigating how carrion disappearance rates are affected by the presence of the two species could be conducted to provide additional clues on the reasons of these avoidance behaviors. In both cases—low individual densities or quicker carrion disappearance—intraspecific and interspecific avoidance would be somewhat similar. Although our study design did not enable individual identification, preventing us from estimating the amount of intraspecific avoidance, other studies in North‐America have suggested a similar avoidance of heterospecifics and conspecifics in these two species (Lai et al., 2022; Rodrigues & Roth, 2023).

The fact that we did not find strong signs of asymmetric competition for carrion between the two species is not necessarily inconsistent with the idea that red foxes limit Arctic foxes' recovery in Fennoscandia. Previous studies that focused on predator interactions around carcasses found that interactions at a short time scale could be very different from the known interactions at the scale of the population. For instance, Mattisson, Andrén, et al. (2011) showed that wolverine populations benefit from coexistence with lynx, and they suggested that the presence of lynx could enhance wolverines reproduction by providing them abundant reindeer carrion. However, despite these effects at the population scale, the same authors found in Mattisson, Persson, et al. (2011) that wolverines tended to avoid direct encounters with lynx to mitigate risks. On the contrary, although wolves (*Canis lupus*) are known to suppress coyotes at the landscape scale (Palomares & Caro, 1999; Tattersall et al., 2020), Sivy, Pozzanghera, Grace, & Prugh (2017) found a positive association between coyotes and wolves at the local scale due to carrion provisioning. In our case, although red foxes are known to negatively affect Arctic fox populations in Fennoscandia, it is possible that different aspects of Arctic and red foxes ecology—such as competition for dens or food in spring—could explain the competitive exclusion at the year‐to‐year scale (Hamel et al., 2013; Rød‐Eriksen et al., 2023). Indeed, the high seasonality of tundra ecosystems in Arctic and alpine areas, combined with a different degree of adaptation to cold and food scarcity between the two species (Hersteinsson & MacDonald, 1982, 1992), make it possible for the patterns of behavioral interactions and interference competition to vary between seasons. For instance, in winter, the lower body condition of red foxes might give Arctic foxes a competitive advantage that decreases during the summer, when conditions get less severe. Red foxes could then develop more aggressive behaviors and monopolize food resources and dens, which could in turn affect breeding success of Arctic foxes.

Availability of live prey is expected to have opposing effects on carrion use. On the one hand, rodent abundance is an important driver of both species' population dynamics on the Varanger Peninsula, where it is known that Arctic foxes only breed in years with high lemming densities (Elmhagen et al., 2017; Ims et al., 2017). On the other hand, as facultative lemming specialists, Arctic foxes are expected to prioritize this prey over carrion whenever possible. The higher colonization of Arctic and red foxes after years with high rodent abundance (Table 1, Figure 6) likely shows that rodents mainly affected carrion use through a bottom‐up increase in both species' population size due to higher prey availability. Therefore, even when other food resources are available, which potentially causes a lower reliance on carrion, foxes keep visiting them, consistent with their known opportunistic behavior. When red foxes were present however, Arctic fox colonization probabilities remained low, regardless of rodent abundance, but the same tendency was not observed in red foxes. As lemming‐specialists, the carcass appeal for Arctic foxes could be lower than for red foxes, especially when other preys are available, making them more likely to switch to rodents than red foxes. The fact that competitive interactions at carrion change when rodents are abundant, with a higher priority for red foxes, may also suggest different competitive abilities between the two species. Red fox is generally described as a dominant species over the Arctic fox due to its bigger size (Elmhagen et al., 2017; Hersteinsson & MacDonald, 1982, 1992), and an encounter might be risky for Arctic foxes. Although we did not find signs for asymmetric competition in the general case, it is possible that Arctic foxes only risk these interactions when their reliance on carrion to survive winter is at the highest, that is, when rodent abundance is at the lowest. It has already been found that the presence of carcasses, when associated with abundant live prey, leads to a resource partitioning between mesopredators (Sivy, Pozzanghera, Colson, et al., 2017; Yarnell et al., 2013). For instance, Sivy, Pozzanghera, Colson, et al. (2017) found that the presence of wolf‐killed carcasses influenced diet composition in red foxes and coyotes, with the bigger and competitively dominant coyote specializing on carcasses, while red foxes kept using rodents, minimizing dietary overlap. They suggested that presence of important carrion supplies could facilitate coexistence between mesopredators by enabling the dominant species to specialize on carcasses. Our results are in accordance with this idea by suggesting that high rodent abundance lead Arctic and red foxes to specialize on different resources, potentially alleviating competition for food. Supplementary feeding—providing Arctic foxes with important additional food supplies unavailable to red foxes—did not appear to affect the outcome of interspecific competition for carrion (Table 1, Figure 6). This is rather surprising as with access to abundant and predictable resources in the area (Ehrich & Ims, 2021), Arctic foxes could have been expected to risk encounters with red foxes to a lesser extent, like they do in years with high rodent abundance. Even though they appear to prioritize lemmings over carrion, they do not seem to prefer the dog pellets used in the feeding stations over the carrion, with the latter possibly being a more profitable food, for which it is worth risking encounters with red foxes. Supplementary feeding associated with reintroduction efforts caused a rapid population increase (Ulvund et al., 2021), incomparable with the year‐to‐year effect of rodent abundance. The profound population increase combined with the supplementary feeding may also have decreased the competitive dominance of red foxes through better body conditions of Arctic foxes next to feeding stations, and through favored group formation due to higher numbers, as it has been shown for coyotes (Tattersall et al., 2020) and suggested for Arctic foxes in other regions (Angerbjörn et al., 2013; Elmhagen et al., 2013; Norén et al., 2012).

We focused on the interactions between Arctic and red foxes as two species known to compete directly (Elmhagen et al., 2017) and the most abundant mammalian predators in our study area, but the carnivore community on the peninsula is in fact more diverse, and the interaction network within the guild of tundra predators is particularly intricate (Rød‐Eriksen et al., 2023). For instance, using a static occupancy model (Rød‐Eriksen et al., 2023), revealed that the presence of wolverines promotes co‐occurrence of Arctic foxes with both red foxes and golden eagles. In our case, considering the whole range of possible interactions within the community might have provided more information about its dynamics at a shorter time scale. However, given the available data, it would have been difficult to fit a more complex model and probably not possible to estimate all parameters. In particular, it is possible that presence of wolverines affected Arctic and red foxes' carrion use in different ways, and modifies their competitive interactions, affecting their ability to co‐occur.

Finally, our model enabled us to estimate the effects of geographical variability on carrion use. We found that Arctic foxes mostly occupied carcasses in tundra farthest from the forest (Table 1, Figure 5), while red foxes seemed to occupy carcasses independently of distance to the forest. This is in accordance with the habitat preference of the two species. Arctic foxes are indeed described as tundra specialists (Alexandra et al., 2002) while red foxes are more generalists and are thus expected to use different habitats to a similar extent (Hersteinsson & MacDonald, 1982). Moreover, the coast to land gradient impacted the two species' carrion use in opposite ways: Arctic foxes used carcasses located inland more than in coastal areas, while red foxes used the coastal areas more (Table 1, Figure 5). Coastal habitats are characterized by important productivity due to marine resources, as well as proximity to human settlements (Killengreen et al., 2011). Red foxes' higher presence next to the coasts probably reflects a preference for these more productive habitats, as it was suggested in previous studies from the Varanger Peninsula (Hamel et al., 2013; Killengreen et al., 2011). The fact that we observed fewer Arctic foxes using carrion close to the coast can be interpreted as a preference for inland habitats, suggesting that they do not use marine or coastal resources on Varanger, unlike in other places of the world where red foxes are absent (Ehrich et al., 2015; Nater et al., 2021; Stickney et al., 2014), which could be due to competitive exclusion, or reflect the geographical range of lemmings. Overall, these results, as well as the higher occupancy next to feeding stations (Table 1, Figures 5 and 6), suggest an important correlation between habitat use and carrion use in Arctic and red foxes.

### 4.1 Model limitations

Our observation model could not fully account for non‐detection because of the mobility, and low abundance of the species studied, which probably resulted in violation of the closure assumption. In occupancy models not accounting for imperfect detection at all can cause the confusion between occupancy and detectability. Hence, an observation model like ours, with systematic lack of fit, is probably better than not accounting for detectability at all (Guillera‐Arroita et al., 2014). Continuous time occupancy models are now starting to be developed (Emmet et al., 2021; Kellner et al., 2022), and they may be good solutions to overcome the difficulties of modeling the detection process for mobile species.

In addition, estimating how species interactions are influenced by environmental drivers requires large amount of data. Despite 16 years of data, we observed a low number of species occurrence, and even less co‐occurrence, which likely caused the large uncertainty in the model estimates. In particular, we chose to use a dynamic framework rather than a static one (Rota et al., 2016), causing the model to require estimation of a large number of parameters. This choice attempted to describe species interaction in a more mechanistic way, but may also have caught confusion in the estimation of the different parameters (e.g., colonization and extinction). We expect that with more years of data, and maybe by increasing the number of camera trapping sites, these uncertainties regarding the estimation of some parameters could be reduced.

## AUTHOR CONTRIBUTIONS

**Simon Lacombe:** Conceptualization (lead); data curation (supporting); formal analysis (lead); writing – original draft (lead); writing – review and editing (equal). **Rolf Ims:** Conceptualization (supporting); formal analysis (supporting); writing – review and editing (equal). **Nigel Yoccoz:** Formal analysis (supporting); writing – review and editing (equal). **Eivind Flittie Kleiven:** Formal analysis (supporting); writing – review and editing (equal). **Pedro G. Nicolau:** Formal analysis (supporting); writing – review and editing (equal). **Dorothee Ehrich:** Conceptualization (supporting); data curation (lead); formal analysis (supporting); supervision (lead); writing – review and editing (equal).

## FUNDING INFORMATION

Data collection and DE were financed by the Norwegian Environmental Agency through the project “COAT arctic fox Varanger.”

## CONFLICT OF INTEREST STATEMENT

We have no conflict of interest to declare.

## Supporting information


Appendix S1:

## Data Availability

The camera trap dataset is publically available on Dryad (https://doi.org/10.5061/dryad.41ns1rnnn). All R scripts used to run the analysis are available on the following Github repository: https://github.com/SimLacombe/TundraFoxes.git.
