# Supplementary material for: Effects of resource availability and interspecific interactions on Arctic and red foxes' winter use of ungulate carrion in the Fennoscandian low‐Arctic tundra
Source: Ecol Evol. 2024 Apr 1;14(4):e11150. doi: 10.1002/ece3.11150 (PMC10985358; doi:10.1002/ece3.11150)
Supplement: Supplementary file 1 — Appendix S1: [file ECE3-14-e11150-s001.pdf]

Supplementary material for: "Effects of resource availability and interspecific interactions on Arctic and red foxes' winter use of ungulate carrion in the Fennoscandian low-Arctic tundra"

Simon Lacombe ✉[1,2], Rolf Ims[2], Nigel Yoccoz[2], Eivind Flittie Kleiven[2,3], Pedro G. Nicolau[2], Dorothee Ehrich[2]

[1] Ecole Normale Superieure de Lyon, Département de Biologie, 69342 Lyon Cedex 07, France Lyon, 69342, FR

[2] UiT The Arctic University of Norway, Department of Arctic and Marine Biology, Framstredet 39 Tromso, Troms, 9037, NO

[3] Norwegian Institute for Nature Research, Framsenteret Tromsø, 9296, NO

Correspondance: Simon Lacombe (simon.lacombe@ens-lyon.fr)

Appendix S1: Two species Dynamic occupancy model

We modelled arctic and red foxes presence at camera sites by fitting a two-species dynamic occupancy model. [1, 2, 3] This model is composed of two parts: an observation model and a transition model.

Observation model

The observation model aims at describing the process by witch species are detected or not at each independent survey (i.e. days), conditionally on the true occupancy state. The following matrix gives the probability of observing each state depending on the true state at the camera trapping site, where $\boldsymbol{\rho^X}$ is the detection probability of species $\boldsymbol{X}$.

$$\text{Observed state}\quad \begin{array}{c|c} & \text{True state} \\ & \begin{array}{cccc} \mathbf{0} & \boldsymbol{A} & \boldsymbol{R} & \boldsymbol{AR} \end{array} \\ \begin{array}{c} \mathbf{0} \\ \boldsymbol{A} \\ \boldsymbol{R} \\ \boldsymbol{AR} \end{array} & \begin{pmatrix} 1 & 1-\rho^A & 1-\rho^R & (1-\rho^A)(1-\rho^R) \\ 0 & \rho^A & 0 & \rho^A(1-\rho^R) \\ 0 & 0 & \rho^R & (1-\rho^A)\rho^R \\ 0 & 0 & 0 & \rho^A\rho^R \end{pmatrix} \end{array} \tag{S1}$$

Transition model

The way the occupancy state varies between two consecutive weeks is modeled as a Markovian process whose transition matrix is given below. We note $\boldsymbol{\gamma}$ and $\boldsymbol{\epsilon}$ as the colonization and extinction probabilities, respectively. $\boldsymbol{\gamma^{X|Y}}$ (or $\boldsymbol{\epsilon^{X|Y}}$) is the colonization (or extinction) probability when the other species is present in either the initial or in the next state and $\boldsymbol{\gamma^X}$ (or $\boldsymbol{\epsilon^X}$) when it is absent.

We also defined $\boldsymbol{\psi^X}$ as the probability that species $\boldsymbol{X}$ is present at a site during the first week of sampling.

$$\begin{array}{c|c} & \text{Initial state} \\ & \begin{array}{cccc} \boldsymbol{0} & \boldsymbol{A} & \boldsymbol{R} & \boldsymbol{AR} \end{array} \\ \text{New state} \begin{array}{c} \boldsymbol{0} \\ \boldsymbol{A} \\ \boldsymbol{R} \\ \boldsymbol{AR} \end{array} & \begin{pmatrix} (1-\gamma^{A})(1-\gamma^{R}) & \epsilon^{A}(1-\gamma^{R|A}) & (1-\gamma^{A|R})\epsilon^{R} & \epsilon^{A|R}\epsilon^{R|A} \\ \gamma^{A}(1-\gamma^{R}) & (1-\epsilon^{A})(1-\gamma^{R|A}) & \gamma^{A|R}\epsilon^{R|A} & (1-\epsilon^{A|R})\epsilon^{R|A} \\ (1-\gamma^{A|R})\gamma^{R} & \epsilon^{A|R}\gamma^{R|A} & (1-\gamma^{A|R})(1-\epsilon^{R}) & \epsilon^{A|R}(1-\epsilon^{R|A}) \\ \gamma^{A|R}\gamma^{R} & (1-\epsilon^{A|R})\gamma^{R|A} & \gamma^{A|R}(1-\epsilon^{R|A}) & (1-\epsilon^{A|R})(1-\epsilon^{R|A}) \end{pmatrix} \end{array} \tag{S2}$$

Covariates

Each parameter can be modelled as a function of a set of covariates using the logit link function. For instance, the colonization probability of species X at time t and site i is defined as follows:

$$logit(\gamma_{t,i}^{X}) = \alpha_{t,i}^{T}\beta_{\gamma}^{X} \tag{S3}$$

Where α_{t_i} is the vector of covariates at time t and site i and β_{γ}^{X} is a vector of intercept and slopes for the colonization probability of species X. To facilitate interpretations, the slope vector for a parameter in the presence of the competitor is defined using the slope vector for the same parameter without the competitor. For instance $\gamma^{X|Y}$ is defined as follows:

$$logit(\gamma_{t,i}^{X|Y}) = \alpha_{t,i}^{T}\left(\beta_{\gamma}^{X} + \beta_{\gamma}^{X|Y}\right) \tag{S4}$$

Note that, in equation (S4), the effect of competition on γ is function of all covariates used to model ψ. Still, it is possible to make competition depend on a subset of these covariates only, by forcing $\left(\beta_{\gamma}^{X|Y}\right)_{C} = \mathbf{0}$ for C the set of all unmodelled covariates.

Appendix S2: Bayesian Goodness of Fit test

To evaluate how the observation and the transition models fit the data, we performed a posterior predictive check [4]. We first simulated 10,000 datasets using the estimated parameters associated with 10,000 randomly selected MCMC iterations. Then, for each of these 10,001 datasets, we calculated for every site (a site being defined as a camera × year combination) the number of detection of each species ($\boldsymbol{Nd_{s,i,o}}$, for species $\boldsymbol{s}$, site $\boldsymbol{i}$ and MCMC step $\boldsymbol{o}$ and $\boldsymbol{Nd_{s,i,dat}}$ for the true dataset) and the number of weeks within each occupancy state ($\boldsymbol{Nw_{m,i,o}}$, for state $\boldsymbol{m}$, site $\boldsymbol{i}$ and MCMC step $\boldsymbol{o}$ and $\boldsymbol{Nw_{s,i,dat}}$ for the true dataset). We also calculated the expected values of $\boldsymbol{Nd}$ ($\widehat{\boldsymbol{Nd}}$) and $\boldsymbol{Nw}$ ($\widehat{\boldsymbol{Nw}}$) for each MCMC iteration. These metrics enabled us to define the following Chi-squared statistics:

$$\begin{aligned}
\chi^2_{DM,sim,o} &= \sum_{i=1}^{N}\sum_{s=1}^{2}\frac{\left(Nd_{s,i,o}-\widehat{Nd}_{s,i,o}\right)^2}{\widehat{Nd}_{s,i,o}}\\
\chi^2_{DM,dat,o} &= \sum_{i=1}^{N}\sum_{s=1}^{2}\frac{\left(Nd_{s,i,dat}-\widehat{Nd}_{s,i,o}\right)^2}{\widehat{Nd}_{s,i,o}}\\
\chi^2_{TM,sim,o} &= \sum_{i=1}^{N}\sum_{m=1}^{4}\frac{\left(Nw_{m,i,o}-\widehat{Nw}_{m,i,o}\right)^2}{\widehat{Nw}_{m,i,o}}\\
\chi^2_{TM,dat,o} &= \sum_{i=1}^{N}\sum_{m=1}^{4}\frac{\left(Nw_{m,i,dat}-\widehat{Nw}_{m,i,o}\right)^2}{\widehat{Nw}_{m,i,o}}
\end{aligned} \tag{S5}$$

Where $\boldsymbol{\chi^2_{\bullet,sim,o}}$ is the chi-squared discrepancy of the simulated dataset and $\boldsymbol{\chi^2_{\bullet,dat,o}}$ the chi-squared discrepancy of the true dataset for MCMC step $\boldsymbol{o}$. $\boldsymbol{TM}$ and $\boldsymbol{DM}$ respectively refer to the transition model and the detection model. $\boldsymbol{N}$ refers to the total number of sites.

Finally for each model, we defined the Bayesian p-value as the proportion of MCMC steps at which the discrepancy of the simulated dataset exceeded the discrepancy of the true dataset ($\boldsymbol{\chi^2_{\bullet,sim,o} > \chi^2_{\bullet,dat,o}}$). This method makes it possible to detect a systematic lack of fit or an overfitting. The model is generally assumed to fit the data properly when the Bayesian p-value is between 0.1 and 0.9[5].

When lack of fit occurred, we plotted the chi-square residuals for each species and site to try to determine possible origins for this lack of fit (Fig. S2).

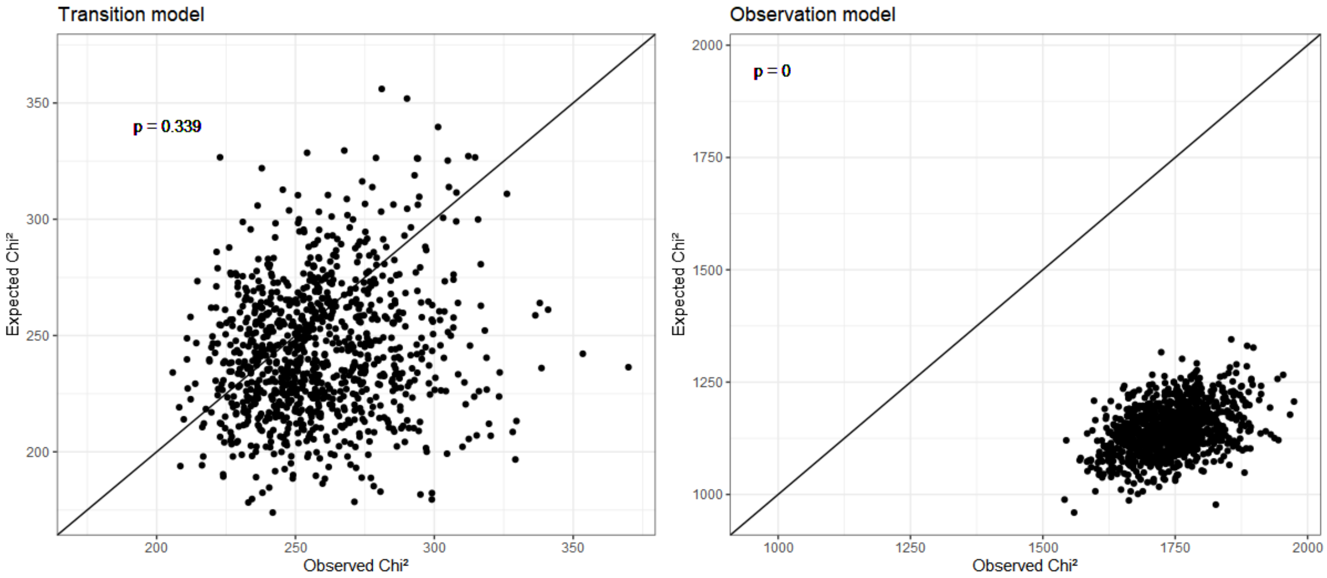

Figure S1: Results of the Bayesian goodness of fit test for the latent and the observation parts of the model. For each MCMC iteration, a dataset was replicated based on the estimated parameters and a χ^2 was calculated for the true and the replicated datasets as a discrepancy measure. The χ^2 value for the simulated dataset is considered the expected value under the hypothesis of a model that perfectly fits the data. Here we show the expected and observed χ^2 values for 10,000 samples of the posterior parameter distribution. The p-values are calculated as the proportion of times st observed $\chi^2 <$ expected χ^2. A model is said to have an appropriate fit for $p \in [0.1, 0.9]$.

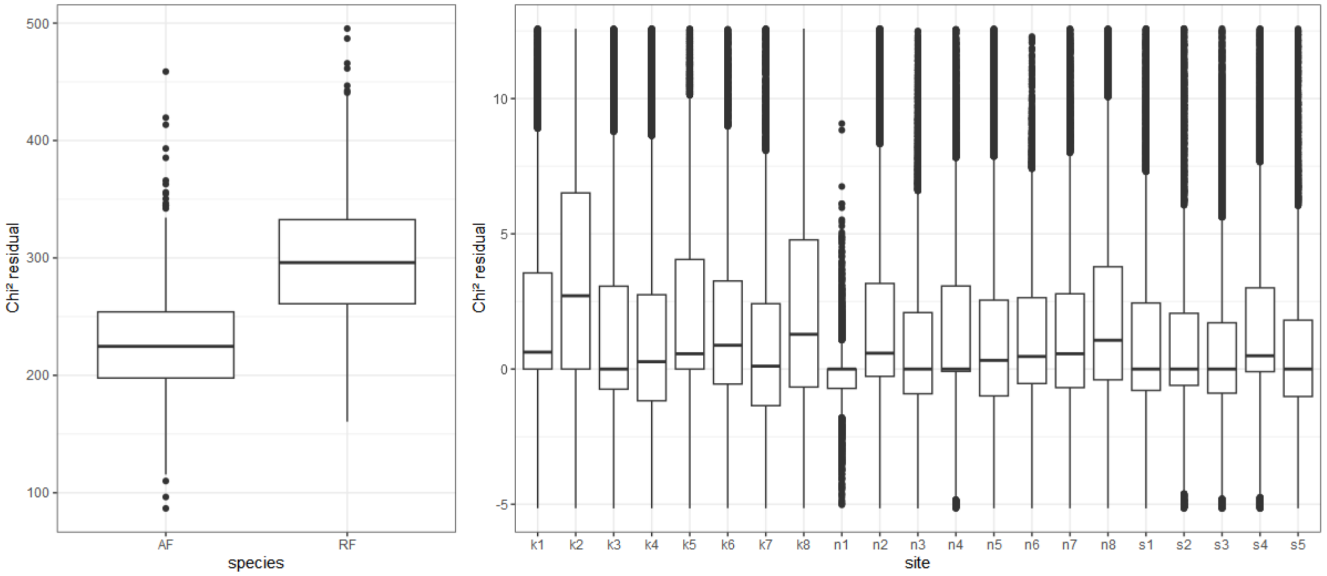

Figure S2: χ_2 residuals for the observation part of the model associated with each species (left panel) and site (right panel).

Appendix S3: Model estimates

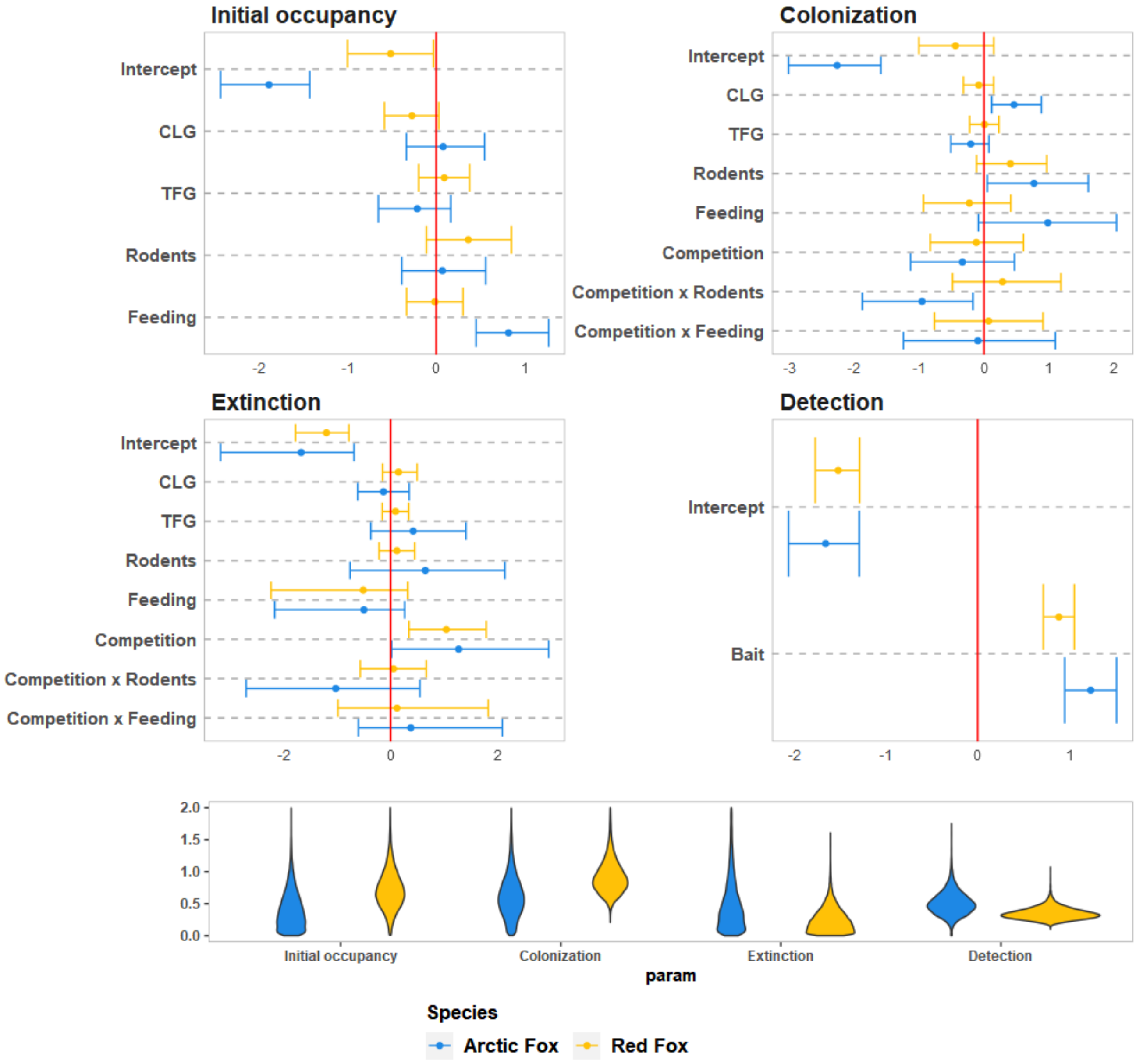

Figure S3: Model estimates. Top panel: 95% credible intervals for the logit-linear intercept and slope parameters. Bottom panel: posterior distribution of the variance associated with random year variation. “CLG” and “TFG” are two geographical gradients and stand for coast to land and tundra to forest gradients, respectively

Appendix S4: PCA on the geographical covariates

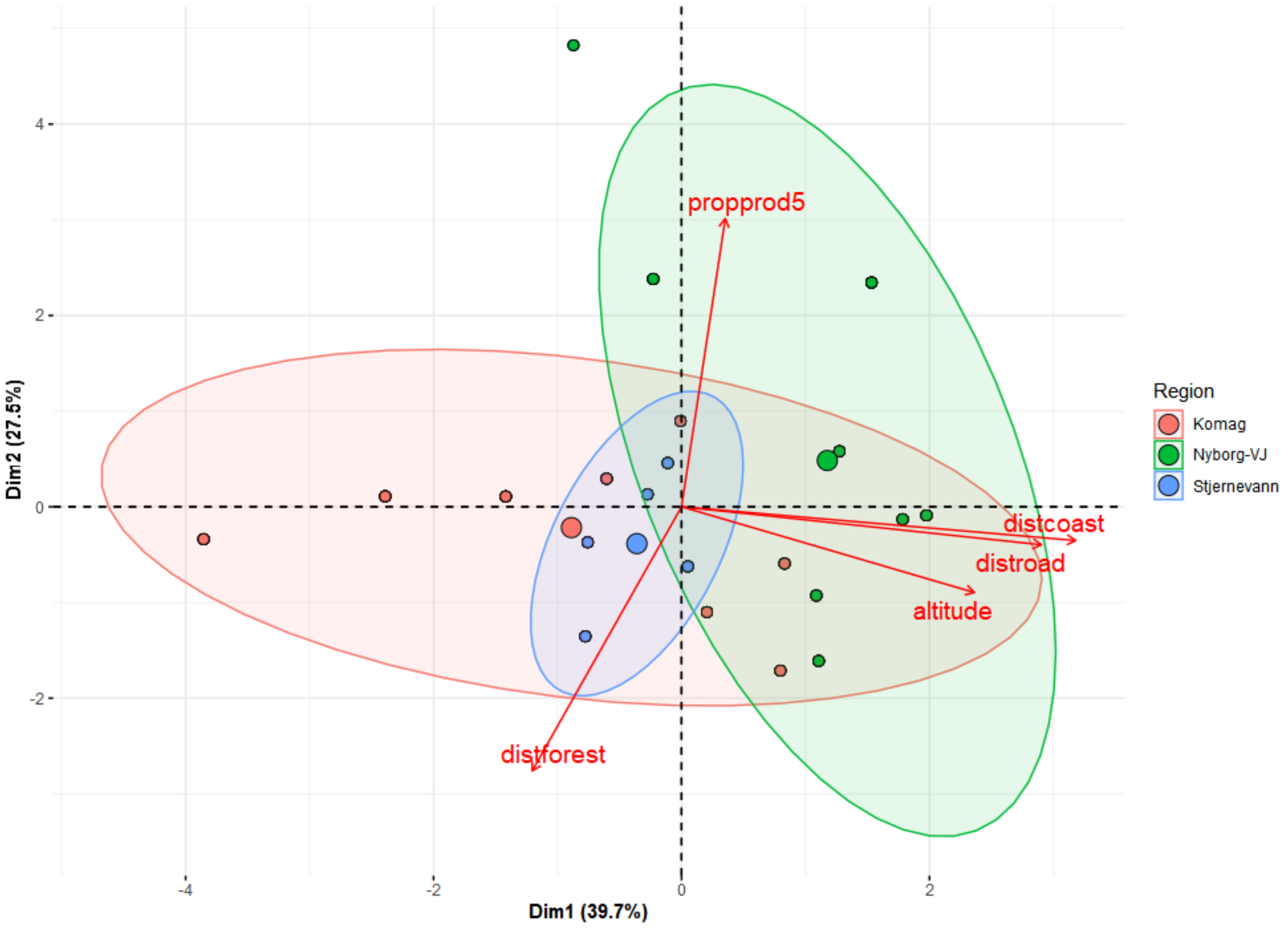

Figure S4: Two first axes of the PCA conducted on the five geographical covariates* measured at the locations of the camera traps. Three sub-regions on Varanger Peninsula are considered (Red: Komagdalen, Green: Vestre-Jakobselv, Blue: Stjernevann). Each of these sub-regions is expected to span across environmental gradients. The contribution of each variable to the two first components are shown with red arrows. These components are then used to define two geographical gradients: Dim1 is identified as a coast to land gradient (CLG) and Dim2 as a tundra to forest gradient (TFG).

*distforest: distance to forest, distroad: distance to road, distcoast: distance to coast, propprod5: proportion of productive areas within a 5km radius.

References

[1] Darryl I. MacKenzie, James D. Nichols, James E. Hines, Melinda G. Knutson, and Alan B. Franklin. ESTIMATING SITE OCCUPANCY, COLONIZATION, AND LOCAL EXTINCTION WHEN a SPECIES IS DETECTED IMPERFECTLY. *Ecology*, 84(8):2200–2207, August 2003.

[2] Christopher T. Rota, Marco A. R. Ferreira, Roland W. Kays, Tavis D. Forrester, Elizabeth L. Kalies, William J. McShea, Arielle W. Parsons, and Joshua J. Millspaugh. A multispecies occupancy model for two or more interacting species. *Methods in Ecology and Evolution*, 7(10):1164–1173, June 2016.

[3] Mason Fidino, Juniper L. Simonis, and Seth B. Magle. A multistate dynamic occupancy model to estimate local colonization–extinction rates and patterns of co-occurrence between two or more interacting species. *Methods in Ecology and Evolution*, 10(2):233–244, November 2018.

[4] EDUARDO CARRILLO-RUBIO, MARC KÉRY, STEPHEN J. MORREALE, PATRICK J. SULLIVAN, BETH GARDNER, EVAN G. COOCH, and JAMES P. LASSOIE. Use of multispecies occupancy models to evaluate the response of bird communities to forest degradation associated with logging. *Conservation Biology*, 28(4):1034–1044, March 2014.

[5] Marc Kery and J. Andrew Royle. *Applied Hierarchical Modeling in Ecology: Analysis of Distribution, Abundance and Species Richness in R and BUGS: Volume 2: Dynamic and Advanced Models*. Academic Press, October 2020.
